# Impact of redox status of donor cows before superovulation treatment on in vivo embryo production

**DOI:** 10.5194/aab-66-433-2023

**Published:** 2023-12-12

**Authors:** Shogo Hashimoto, Masayasu Taniguchi, Ayane Edo, Tetsushi Ono, Tetty Barunawati Siagian, Hiroaki Sekine, Megumi Nagahara, Takeshige Otoi, Mitsuhiro Takagi

**Affiliations:** 1 Joint Faculty of Veterinary Medicine, Yamaguchi University, 753-8515 Yamaguchi, Japan; 2 College of Vocational Studies, Bogor Agricultural University, Bogor, Indonesia; 3 Ranch-related Business Department, Morinaga Dairy Service Corporation, 329-3224 Tochigi, Japan; 4 Bio-Innovation Research Center, Tokushima University, 779-3233 Tokushima, Japan

## Abstract

This study investigated the relationship between oxidation and antioxidation parameters before superovulation (SOV) treatment and embryo recovery in donor cows. The relative redox status of the 61 donor cows was evaluated based on the median values of diacron-reactive oxygen metabolites (d-ROMs) and biological antioxidant potential (BAP) measurements (d-ROMs of 100 U.CARR; BAP of 2413 µ mol L ${}^{-1}$ ) before SOV treatment. Following this, the animals were divided into four groups: cows with low d-ROMs but high BAP were assigned to the “LH” group ( n=11 ), cows with high d-ROMs and BAP were assigned to the “HH” group ( n=20 ), cows with high d-ROMs but low BAP were assigned to the “HL” group ( n=10 ), and cows with low d-ROMs and BAP were assigned to the “LL” group ( n=20 ). Embryos were collected from superovulated cows 6 d after artificial insemination. The percentages of fertilised, transferable, and Code-1 embryos were significantly higher ( P<0.05 ) in the HH group than those in the HL group. The HH group had the highest mean values for all embryo recovery results. Our results suggest that the redox status of donor cows before SOV treatment affects embryo recovery, as cows with high levels of both oxidative and antioxidative status have better embryo production.

## 1 Introduction

Recently, a combination of in vivo embryo production using superovulation (SOV) and embryo transfer has enabled efficient calf production and has dramatically accelerated livestock improvement and breeding. In the field of in vivo embryo production, the relationship between the redox status of donor cows has been studied, and the results suggest that oxidative stress negatively affects in vivo embryo production (Alkan et al., 2021; Karasahin et al., 2021). Oxidative stress manifests as an imbalance between the number of reactive oxygen species (ROS) and the antioxidant capacity of the body (Abuelo et al., 2015; Mandelker, 2008). It has been suggested that oxidative stress may adversely affect reproductive processes, such as oocyte maturation, folliculogenesis, ovulation, steroidogenesis, embryo development, and luteolysis (Guérin et al., 2001; Rizzo et al., 2012). Therefore, clarifying the effects of oxidative stress on embryo production could also help predict donor cow selection and SOV responses.

Malondialdehyde (MDA), a major marker and product of lipid peroxidation, was measured to assess the oxidative status in vivo (Castillo et al., 2006; Tsuchiya et al., 2020). In addition, the antioxidants superoxide dismutase (SOD) and reduced glutathione (GSH) were analysed to evaluate their antioxidant capacities (Abd Ellah et al., 2009; Skaperda et al., 2021; Zhang et al., 2023). However, when the conventional method is used to separately evaluate oxidative stress values and antioxidant capacity, it is uncertain whether individuals with high oxidative stress values are truly in a state of oxidative stress (Lichtenberg et al., 2018), and the measurement is laborious. Recently, diacron-reactive oxygen metabolite (d-ROM) and biological antioxidant potential (BAP) tests have been developed using a free-radical analyser, allowing for easy and rapid evaluation of the oxidative and antioxidative status of a living body from blood samples (Abuelo et al., 2013; Cesarone et al., 1999). The d-ROM test measures oxidative stress levels, primarily by targeting hydroperoxides (ROOH), which are produced when organic matter is oxidised. ROOH is an indicator of current oxidative stress levels and possible future oxidative stress; in addition to arising from organic matter oxidised by ROS, ROOH can be converted into ROS under certain conditions (Fontecave and Pierre, 1993), such as in the presence of metal ions, to oxidise other organic matter. The BAP test measures the overall antioxidant capacity in vivo by measuring the decolourisation intensity of antioxidants in a sample and the reduction of iron ions (Benzie and Strain, 1996). From this, it is possible to calculate the oxidative stress index (OSI), which represents the balance between the oxidative stress level in the blood and the antioxidant capacity, thereby enabling the evaluation of the overall oxidative stress level in the body (Abuelo et al., 2013).

The aim of this study was to investigate the relationship between the donor redox status, assessed by the d-ROMs and BAP measurements, and subsequent embryo recovery results to improve the efficiency of in vivo embryo production by assessing donor cattle before SOV treatment.

## 2 Materials and methods

### 2.1 Donor cows

Between August 2019 and December 2021, 61 healthy Japanese Black multiparous cows (average age ± SD of 48.6 ± 21.6 months; average parity ± SD of 2.1 ± 1.2) were included as donors in this study. All experimental cows were raised using a free-burn system on the same farm and were fed according to Japanese beef cattle feeding standards.

### 2.2 SOV treatment and sample collection

Follicular waves were synchronised by the insertion of a controlled intravaginal drug release device (CIDR, CIDR1900, Zoetis Japan, Tokyo, Japan) and an intramuscular injection of 2 mg of oestradiol benzoate (Ovahormon^®^ injection, Asuka Animal Health, Tokyo, Japan) during the oestrous cycle. SOV treatment was performed using one of the following two methods: (1) the multiple-shot method – 3 AU (armour units) of follicle-stimulating hormone (FSH; Antrin R10, Kyoritsu Seiyaku, Tokyo, Japan) was administered intramuscularly twice daily for a total of seven times; (2) the one-shot method – a single subcutaneous injection of extended-release FSH mixed with aluminium hydroxide gel (Antrin R10/AL; Kyoritsu Seiyaku) was administered. On day 3 of each SOV programme, the cows received cloprostenol (0.5 mg) (Dalmazin^®^, Kyoritsu Seiyaku, Tokyo, Japan) to induce regression of the corpus luteum, and they were subsequently artificially inseminated 60–72 h later. Embryos were collected 6 d after artificial insemination (AI). The recovered embryos were evaluated according to the International Embryo Technology Society criteria (Robertson and Nelson, 1998). Embryos were classified as Code-1 embryos, Code-2 embryos, degenerated embryos, and unfertilised oocytes. Code-1 and Code-2 embryos were defined as transferable embryos, while Code-1, Code-2, and degenerated embryos were defined as fertilised embryos. The percentages of Code-1, transferable, and fertilised embryos in the total recovered embryos/oocytes were calculated.

Before starting SOV treatment in the morning (around 09:00 JST, Japan Standard Time), blood samples were collected from the tail vein in a heparin vacuum collection tube. Plasma was collected after centrifugation at 2000 g for 10 min and stored at -30 ${}^{\circ }$ C until measurement of d-ROMs and BAP.

### 2.3 Measurement of d-ROMs, BAP, and OSI

Using a free-radical analyser (Free Carrio Duo, Wismerll, Tokyo, Japan), d-ROM and BAP tests were performed following the procedure described by Imai et al. (2021), and the OSI (OSI = d-ROMs / BAP × 100) was calculated from the values obtained.

To measure the plasma levels of d-ROMs, 20 µ L of the sample was added to a cuvette (Wismerll) containing 1.2 mL of acidic buffer (pH 4.8) with iron ions, followed by the addition of 20 µ L of d-ROM colouring solution containing N , N -diethyl- p -phenylenediamine, and mixed by inversion. Optical measurements were performed at a wavelength of 505 nm using a free-radical analyser to determine the hydroperoxide (ROOH) concentration. The unit of measurement is expressed in U.CARR, with one U.CARR corresponding to 0.08 mg H ${}_{2}$ O ${}_{2}$ dL ${}^{-1}$ .

To measure the plasma levels of BAP, 50 µ L of BAP colouring solution containing trivalent iron salt (Wismerll, Tokyo, Japan) was added to a cuvette containing 1.0 mL of thiocyanate and mixed by inversion; the absorbance was then measured at a wavelength of 505 nm using a free-radical analyser. After the measurement, 10 µ L of the sample was added and the cuvette was mixed by inversion. The absorbance was measured again, and the change in absorbance was calculated and expressed in micromoles per litre ( µ mol L ${}^{-1}$ ) of reduced ferric ions.

### 2.4 Donor cow groups according to the d-ROMs and BAP levels

**Figure 1:**
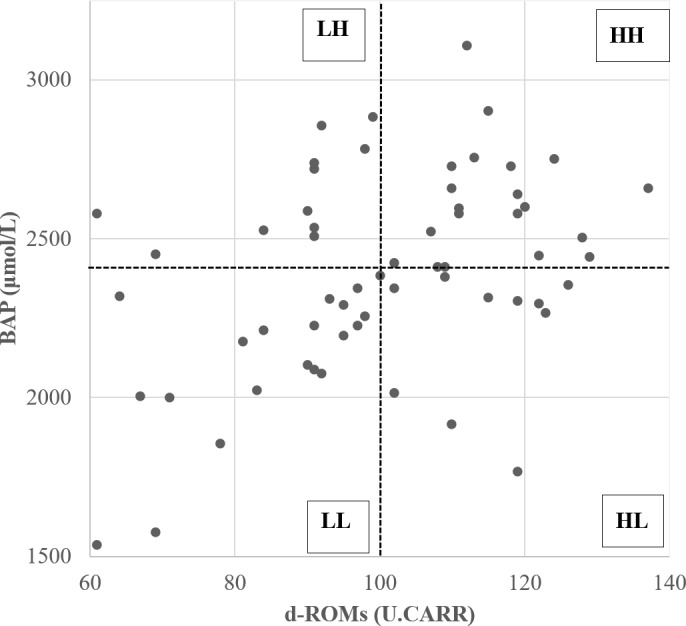
Scatterplot of diacron-reactive oxygen metabolite (d-ROM) and biological antioxidant potential (BAP) values before the start of superovulation treatment. Dotted lines represent the median d-ROMs and BAP values. The abbreviations used in the figure are as follows: LH – low d-ROM and high BAP values; HH – high d-ROM and high BAP values; HL – high d-ROM and low BAP values; and LL – low d-ROM and BAP values.

The relative redox status of the 61 donor cows was evaluated based on the median values of d-ROM and BAP measurements (d-ROM of 100 U.CARR; BAP of 2413 µ mol L ${}^{-1}$ ) before SOV treatment, after which they were divided into four groups (Fig. 1). Table 1 shows the mean values of oxidative status biomarkers in the four donor groups, divided based on the median values of d-ROMs, BAP, and OSI. Cows with low d-ROMs but high BAP were assigned to the “LH” group ( n=11 ), cows with high d-ROMs and BAP were assigned to the “HH” group ( n=20 ), cows with high d-ROMs but low BAP were assigned to the “HL” group ( n=10 ), and cows with low d-ROMs and BAP were assigned to the “LL” group ( n=20 ). The embryo recovery results were compared among the four groups.

**Table 1:** Mean ± SEM (standard error of the mean) of diacron-reactive oxygen metabolites (d-ROMs), biological antioxidant potential (BAP), and oxidative stress index (OSI) before the start of superovulation treatment.

Group ${}^{1}$	No. of	d-ROMs	BAP	OSI ${}^{2}$
	cows	(U.CARR) ${}^{2}$	( µ mol L ${}^{-1}$ ) ${}^{2}$	
LH	11	87.0 ± 3.5 ${}^{a}$	2651 ± 45 ${}^{a}$	3.3 ± 0.1 ${}^{a}$
HH	20	116.2 ± 1.9 ${}^{b}$	2623 ± 40 ${}^{a}$	4.4 ± 0.1 ${}^{b}$
HL	10	114.7 ± 2.7 ${}^{b}$	2195 ± 68 ${}^{b}$	5.3 ± 0.2 ${}^{c}$
LL	20	84.9 ± 2.8 ${}^{a}$	2110 ± 52 ${}^{b}$	4.0 ± 0.1 ${}^{d}$

${}^{1}$ The abbreviations used in the table are as follows: LH – low d-ROM and high BAP values; HH – high d-ROM and high BAP values; HL – high d-ROM and low BAP values; and LL – low d-ROM and BAP values. ${}^{2}$ Data are expressed as the mean ± SEM. ${}^{\text{a–d}}$ Values with different superscript letters in the same column differ significantly ( P<0.01 ).

### 2.5 Statistical analysis

Percentage data were subjected to arcsine transformation prior to analysis. The data for d-ROMs, BAP, OSI, collected ova and fertilised embryos, transferable embryos, and Code-1 embryos were analysed by analysis of variance using the general linear model procedure of SAS (SAS for Windows, version 9.1; SAS Institute Japan, Tokyo, Japan). Analysis of variance was applied to obtain factors for adjustment to age, parity, season, and SOV treatment. Data are expressed as the means ± SEM (standard error of the mean). Statistical significance was set at P<0.05 .

## 3 Results

Age, parity, season, and SOV treatment had no significant influence on the oxidative/antioxidative biomarker levels and embryo recovery results, except that SOV treatment had an effect on the percentages of fertilised embryos (Table 2). The percentages of fertilised, transferable, and Code-1 embryos in the HH group were significantly higher ( P<0.05 ) than those in the HL group (Table 3). Moreover, a significantly higher percentage of transferable embryos was observed in the HH group than in the LH group ( P<0.05 ). The HH group showed the highest mean value for all embryo recovery results.

**Table 2:** Effects of age, parity, season, and superovulation (SOV) treatment on the oxidative/antioxidative biomarker levels and embryo recovery results.

Item ${}^{*}$	Effect, P value			
	Age	Parity	Season	SOV treatment
d-ROMs	0.271	0.904	0.807	0.874
BAP	0.378	0.788	0.532	0.975
Total no. of oocytes/embryos	0.27	0.772	0.159	0.612
Fertilised embryos (%)	0.33	0.366	0.067	0.033
Transferable embryos (%)	0.774	0.842	0.756	0.384
Code-I embryos (%)	0.656	0.528	0.903	0.694

${}^{*}$ Diacron-reactive oxygen metabolites (d-ROMs), biological antioxidant potential (BAP), oxidative stress index (OSI).

**Table 3:** Comparison of embryo recovery results among the four groups based on median values of diacron-reactive oxygen metabolites (d-ROMs) and the biological antioxidant potential (BAP) parameter before superovulation treatment.

Group ${}^{1}$	No. of	Total no. of	Fertilised	Transferable	Code-I
	cows	oocytes/embryos ${}^{2}$	embryos (%) ${}^{2}$	embryos (%) ${}^{2}$	embryos (%) ${}^{2}$
LH	11	18.7 ± 2.9 ${}^{a,b}$	91.0 ± 4.6 ${}^{a}$	41.0 ± 8.3 ${}^{a}$	31.6 ± 6.8 ${}^{a,b}$
HH	20	19.1 ± 2.4 ${}^{a}$	93.7 ± 2.5 ${}^{a}$	62.7 ± 6.7 ${}^{b}$	39.6 ± 4.7 ${}^{a}$
HL	10	12.7 ± 2.0 ${}^{a,b}$	73.6 ± 7.7 ${}^{b}$	33.5 ± 11.0 ${}^{a}$	14.2 ± 5.6 ${}^{b}$
LL	20	12.8 ± 1.7 ${}^{b}$	87.1 ± 3.5 ${}^{a,b}$	55.0 ± 5.2 ${}^{a,b}$	38.9 ± 5.2 ${}^{a}$

${}^{1}$ The abbreviations used in the table are as follows: LH – low d-ROM and high BAP values; HH – high d-ROM and high BAP values; HL – high d-ROM and low BAP values; and LL – low d-ROM and BAP values. ${}^{2}$ Data are expressed as the mean ± SEM. ${}^{\text{a–b}}$ Values with different superscript letters in the same column differ significantly ( P<0.05 ).

## 4 Discussion

The ROS/antioxidant balance in animals is critical for oocyte maturation, follicular atresia, fertilisation, embryo development, maintenance, and regression of luteal tissue (Guérin et al., 2001; Rizzo et al., 2012). In this study, donor cows were divided based on the median values of d-ROMs and BAP before SOV treatment, and embryo recovery results were evaluated. Classification based on median values appeared appropriate because there were significant differences between the two groups for each oxidative/antioxidative biomarker level, and the biomarker levels were not significantly affected by age, parity, or season. As a result, higher proportions of transferable and Code-1 embryos were observed in the HH group than in the HL group. These results indicate that donor cows with both a high oxidative and antioxidative status before SOV treatment improve in vivo embryo production.

In the present study, we expected that the LH group, with a low oxidative status and high antioxidative status, would have better embryo recovery results than the other groups. However, our results showed that the proportion of transferable embryos was lower in the LH group than in the HH group. Moreover, the HH group had the highest mean value among the four respective donor groups. These results suggest that low oxidative status may not always be favourable for reproduction in cattle. Although ROS are toxic to living organisms and cause various types of damage, including DNA fragmentation, membrane disruption, and enzyme inactivation, appropriate an number of ROS also function as intracellular signalling devices by altering the redox state of target proteins, such as translation control factors (Fujii et al., 2005). The importance of ROS has also been reported in the field of reproduction, where they regulate steroid hormone secretion in the ovary (Carlson et al., 1993; Fujii et al., 2005; Sawada and Carlson, 1996), ovulation (Fujii et al., 2005; Shkolnik et al., 2011), and sperm maturation (Fujii et al., 2005), and promote the binding of the zona pellucida (Sanocka and Kurpisz, 2004). It has also been suggested that follicular cysts in cattle due to ovulation failure are associated with low ROS concentrations in the follicular fluid (Rizzo et al., 2009). Reactive nitrogen species (RNS), often classified as ROS, have also been shown to be important for ovulation and oocyte maturation. Jablonka-Shariff et al. (1999) treated mice with superovulation and subsequently orally administered an inhibitor of nitric oxide, an RNS, and observed that oocyte maturation was then blocked. Therefore, it is possible that the low oxidative status of cows in the LH group in the present study might have had negative effects on processes such as ovulation and oocyte maturation.

In summary, the redox status of donor cows prior to SOV treatment may affect embryo recovery. Moreover, evaluation of the redox status of donor cows may predict embryo recovery results before SOV. This information will be useful for the selection of donor cows and subsequent treatments.

## Data Availability

The data sets used in this paper are available from the corresponding author upon request.
